# Axial loading and posture cues in contraction of transversus abdominis and multifidus with exercise

**DOI:** 10.1038/s41598-020-67509-1

**Published:** 2020-07-14

**Authors:** Patrick J. Owen, Timo Rantalainen, Richard A. Scheuring, Daniel L. Belavy

**Affiliations:** 1 Institute for Physical Activity and Nutrition, School of Exercise and Nutrition Sciences, Deakin University, Geelong, VIC 3220 Australia; 2 Gerontology Research Center and Faculty of Sport and Health Sciences, University of Jyväskylä, Jyväskylä, Finland; 3 Johnson Space Center, 2101 NASA Parkway SD4, Houston, TX 77058 USA

**Keywords:** Physiology, Musculoskeletal system

## Abstract

Astronauts are at increased risk of spine injury. With a view to developing training approaches for the muscles of the spine in microgravity, this study examined the effects of axial loading and postural cues on the contraction of transversus abdominis and lumbar multifidus in supine lying using a novel exercise device (GravityFit). Thirty (18 males and 12 females) endurance-trained runners without a history of spinal pain aged 33–55 years were recruited. Magnetic resonance imaging (MRI) was performed under one rest and five exercise conditions, which involved variations in axial loading and postural cues. Whole volume of the abdominal and lumbar paraspinal muscles was imaged and transversus abdominis thickness and length and multifidus anteroposterior and mediolateral thickness measured. Transversus abdominis contraction was greatest in the ‘stretch tall plus arm extension’ (length, − 15%, P < 0.001; thickness, + 19%, P < 0.001) and ‘stretch tall plus arm extension and thoracic cue’ (length, − 16%, P < 0.001; thickness, + 18%, P < 0.001) conditions. The contraction of multifidus was the greatest in the ‘arm extension and thoracic cue’ (anteroposterior, + 3.0%, P = 0.001; mediolateral, − 4.2%, P < 0.001) and ‘stretch tall plus arm extension and thoracic cue’ (anteroposterior, + 6.0%, P < 0.001; mediolateral, − 2.1%, P = 0.022) conditions. This study provides proof-of-principle for an exercise approach that may be used to facilitate the automatically contraction of the transversus abdominis and multifidus muscles. Axial loading of the body, with or without arm loading, most consistently led to contraction of the transversus abdominis and lumbar multifidus muscles, and regional differences existed in the contraction within the muscles.

## Introduction

With a long-term view to missions to Mars or a Moon base, amelioration of musculoskeletal deterioration is important to ensure success of mission tasks and to safeguard return to Earth. Astronauts are at increased risk of lumbar spine injury^1^ and muscular deficiencies have been suggested as a likely risk factor^2^. The extensor musculature of the spine^3^, and in particular the lumbar multifidus^4^, are important for controlling the lumbar spine. Recent data from six astronauts showed that multifidus muscle atrophy persisted 6 weeks after spaceflight^5^. Additionally, the deep antero-lateral abdominal muscles, such as the transversus abdominis, are considered to stabilise the lumbar spine^6^. Via the thoracolumbar fascial connection to the lumbar spine, contraction of these muscles increases lumbar spine stiffness^6^. Moreover, intra-abdominal pressure plays a role in modulating lumbar spine stiffness and therefore stability. Transversus abdominis muscle contraction increase intra-abdominal pressure^7^, and thus lumbar spine stability^8^. Furthermore, stiffness of the sacroiliac joint is increased by contraction of the deep antero-lateral abdominal muscles^9^. In spaceflight simulation (i.e. prolonged bed-rest), atrophy of the transversus abdominis has been observed^10^. Therefore, when the musculature in microgravity, it is critical that exercise training approaches are independent of gravity.

This study aimed to examine the effects of axial loading and postural cues whilst using a novel gravity-independent exercise device on transversus abdominis and multifidus contraction. Contraction of the muscles of the trunk can be measured via magnetic resonance imaging (MRI)^11,12^. One advantage of techniques such as MRI (and ultrasound)^13–16^ for the assessment of trunk muscles is that the deep musculature, such as transversus abdominis, can be assessed without invasive techniques such as fine-wire electromyography. Contraction of transversus abdominis, as assessed by structural imaging, is exemplified by anteroposterior shortening and thickening of this muscle^11^. Contraction of multifidus is associated with increases in its thickness^15^. Furthermore, structural changes in the trunk muscles with contraction assessed via imaging techniques, such as thickness increases of the transversus abdominis muscles^13,14,16^ and multifidus muscle^15^, correlates with electromyographic activity. The primary hypothesis was that axial loading and specific postural cues, as assessed via MRI, would result in the contraction of the transversus abdominis and multifidus compared to rest.

## Methods

### Participants and study details

This was a cross-sectional study^17^ conducted from September 2017 to December 2017 at a medical imaging centre in Melbourne, Australia. The study was approved by Deakin University Faculty of Health Human Ethics Advisory Group. All participants gave their informed written consent prior to participation. This research complied with the Australia Code for the Responsible Conduct of Research (2018). Participants were provided with a pre-paid money card worth A$60 to remunerate their time/travel expenses.

A collective was recruited to reflect the current United States National Aeronautics and Space Administration (NASA) Astronaut Corps. Endurance-trained males and females aged 33–55 years were included in the study on a ratio of 6:4. Individuals were deemed endurance-trained if they participated in at least one half-marathon (approx. 21 km) distance run in the past year and trained at least twice a week for running for the last 1.5 years or greater. Thirty participants were recruited by online advertisement (Facebook) and direct contact with running clubs in Victoria, Australia. Exclusion criteria included: (1) regular training for other sports more than one day per week within the last year, (2) prior participation in high level sporting codes known to impact spine health (i.e. volleyball, swimming, water polo, weight lifting, rowing, cricket [as a bowler], baseball, gymnastics, American football, equestrian or wrestling), (3) current shoulder, thoracic, neck or lumbar spine pain for which treatment was sought (‘treatment’ was defined as having seen a physiotherapist, chiropractor, osteopath or medical doctor for the condition), (4) history of shoulder, thoracic, neck or lumbar spine pain for which more than one treatment session was sought, (5) known scoliosis or osteoporosis, and (6) unable to communicate in English. Absolute contraindications to MRI were also implemented (e.g. metal or electrical implants, claustrophobia or possible pregnancy).

### Testing and exercise protocol

Participants were instructed to avoid exercise on the day of testing. Upon arriving at the medical imaging facility, participants completed questionnaires detailing their demographics. Height and weight were measured using a portable stadiometer and scales.

**Figure 1:**
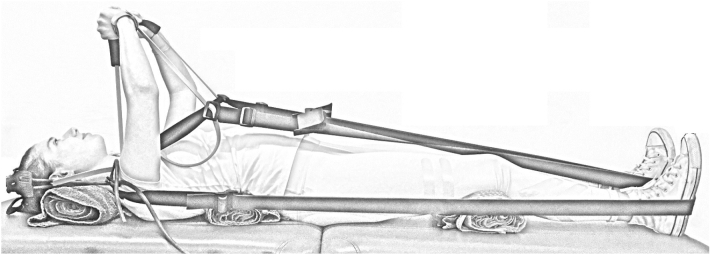
Image of the GravityFit exercise apparatus during compression through the body and arms. Example of condition four (stretch tall plus arm extension and thoracic cue).

A resistance band-based exercise device designed to provide axial loading through the spine and lower-limbs, as well as the arms during arm extension was used (GravityFit, Peregian Beach, Australia; www.gravityfit.com; Fig. 1). The resistance of the bands was an estimated 20% one-repetition maximum based on the threshold between ‘average’ and ‘good’ normative values for age, sex and weight^18^. Resistance was determined by digital force gauge (Digital Scale 40 kg, Rogue, Lawnton QLD, Australia). MRI was performed under six conditions, which involved variations in loading and postural cues in the following sequence: (1) rest (no loading or cues provided; control 1: no equipment), (2) stretch tall only (axial loading through the length of the body with stretch tall cue to avoid slouching whilst using equipment; control 2: equipment only), (3) stretch tall plus arm extension (axial loading through the length of the body and arms with stretch tall cue; experimental condition 1), (4) stretch tall plus arm extension and thoracic cue (axial loading through the length of the body and arms with stretch tall and thoracic cue; experimental condition 2), (5) arm extension only (axial loading through arms with no cues provided; experimental condition 3) and (6) arm extension and thoracic cue (axial loading through arms with stretch tall and thoracic cue; experimental condition 4). Prior to the scanning protocol, postural cues were explained to participants. The ‘stretch tall cue’ was explained as slowly elongating body length from the feet through to the crown of the head, which aimed to lengthen the spine and avoid slouching posture given the equipment examined. The ‘thoracic cue’ was explained with the following instructions: ‘tuck your chin in’ (drawing the head directly backwards whilst maintaining level head position, which aimed to relax neck and shoulder musculature), ‘relax shoulders down and push into the foam whilst keeping shoulder blades on the bed’ (pushing the thoracic spine out against a piece of foam placed between the retracted scapulae whilst relaxing the shoulder musculature, which aimed to activate the thoracic core). Rolled towels under the neck and lumbar spine were used to maintain a neutral spine position and a rolled towel was positioned under the knees to prevent knee straightening. During arm extension, participants were asked to maintain an isometric arm extension during 30-s scans. During all conditions, participants were instructed to hold their breath and remain static during scans.

### Magnetic resonance imaging, image processing and analysis

To quantify muscle morphology on a 3 T Phillips Ingenia scanner (Amsterdam, Netherlands) a T2-weighted sequence (thickness, 3 mm; interslice distance, 7 mm; repetition time, 2,643 ms; echo time, 60 ms; field of view, 347 × 347 mm, 768 × 768 pixels) was used with spinal coils to collect 14 axial images encompassing the volume of the transversus abdominis and multifidus from the perineum up to the rib cage. Data were exported for offline processing. To ensure blinding of the examiner, each subject was assigned a random numeric code (obtained from www.random.org). ImageJ 1.48v (https://rsb.info.nih.gov/ij/) was used to perform all quantitative MRI measures.

**Figure 2:**
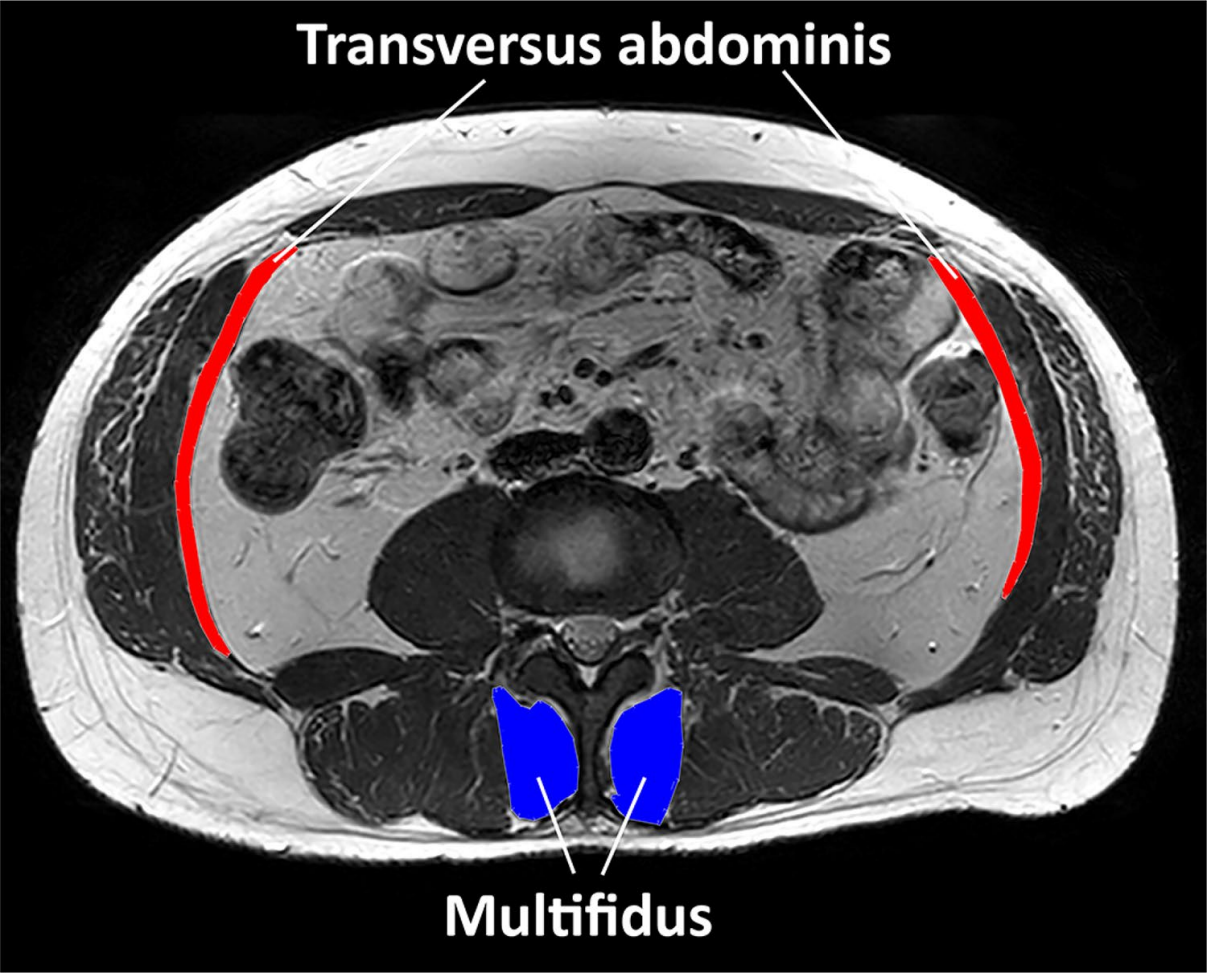
Manual tracing of transversus abdominis (red) and multifidus (blue).

After tracing around the transversus abdominis muscle (Fig. 2), a custom written ImageJ plugin (“ROI Analyzer”; https://github.com/tjrantal/RoiAnalyzer and https://sites.google.com/site/daniellbelavy/home/roianalyser) was used to fit a fourth order polynomial to the region of interest and the curvature from the muscle was removed. Muscle length and thickness were calculated. When multifidus was traced around (Fig. 2), anteroposterior and mediolateral thicknesses were calculated without rotation of the region of interest. The vertebral level present in each image was noted.

Data were then averaged across all slices, as well as between the left and right sides. Similar values were then calculated different regions of the muscle based on the following lumbar and sacral vertebral levels: upper (L1–2), middle (L3–4) and lower (L5–S1). The primary analysis focussed on peak length and mean thickness of transversus abdominis and peak anteroposterior and peak mediolateral thickness of multifidus. The reliability of the outcome measure in our laboratory is excellent, with an ICC_2,1_ of 0.95 for muscle thickness and 0.93 for muscle length.

### Statistical analyses

The “R” statistical environment (version 3.4.2, www.r-project.org) was used for all statistical analyses. A linear-mixed effects model with allowances for heterogeneity of variance according to ‘contraction state’ were performed. Then repeated-measures analysis of variance examined for differences between ‘contraction state’. Then a priori T-tests were performed comparing each contraction state to the first ‘rest’ state. To assess regional differences in muscle contraction, data were converted to percentage change compared to the baseline ‘rest’ condition. Then similar linear-mixed effects models were used to, for each contraction state, compare differences in the percentage change of each parameter between the upper, middle and lower portions of the muscles. An alpha-level of 0.05 was taken for statistical significance. To control for potential type I errors, p-values were adjusted via the false discovery rate method^19^. Adding age, body mass and sex covariates to the models does not change the findings of the study.

## Results

Thirty participants (18 males and 12 females) were measured. Mean (standard deviation) age, height, weight and body mass index were 43 years (7 years), 170.7 cm (9.0 cm), 67.9 kg (10.9 kg) and 23.2 kg/m^2^ (2.5 kg/m^2^), respectively.

### Whole muscle contraction in each manoeuvre

**Figure 3:**
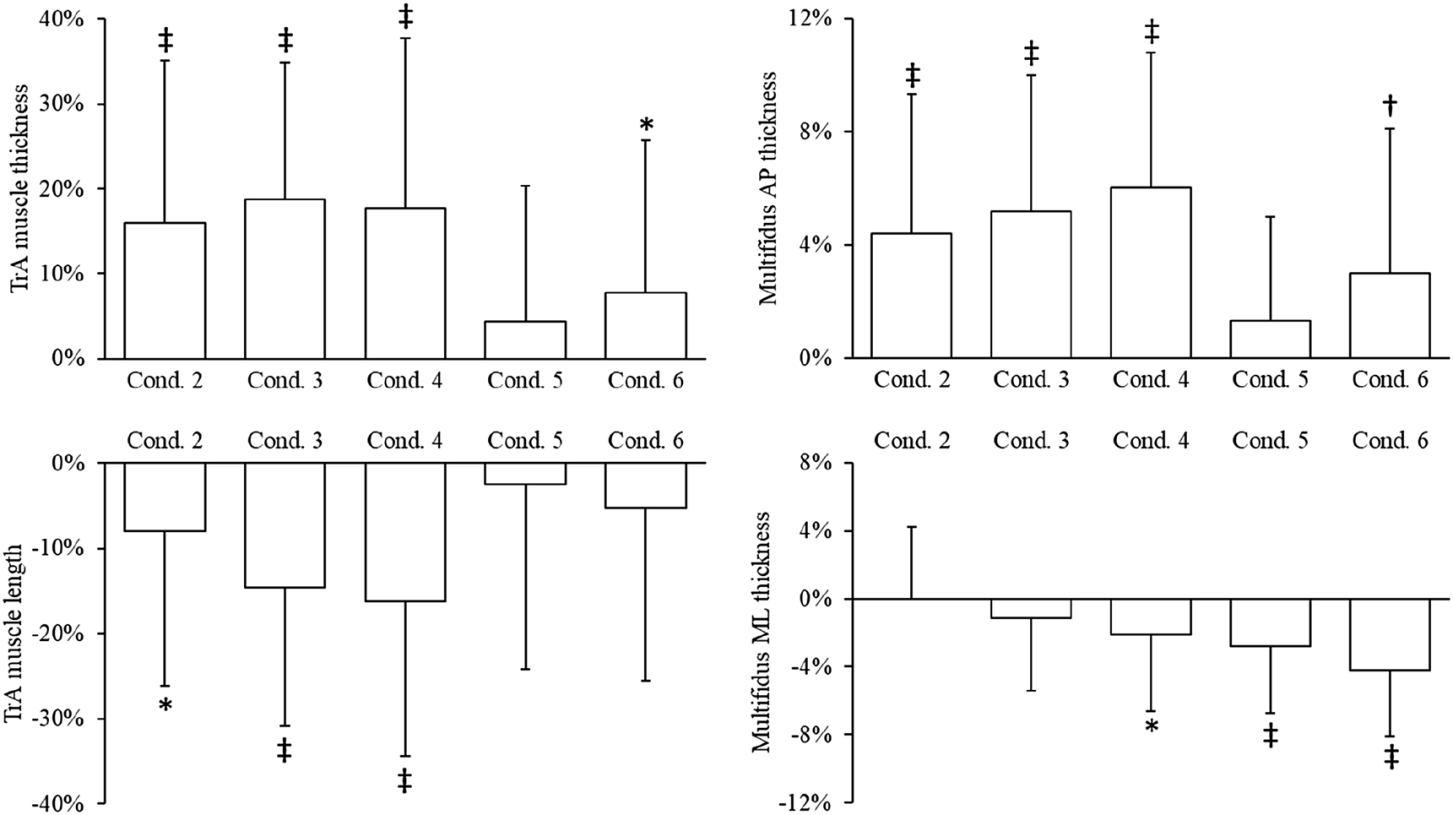
Changes in whole transversus abdominis (TrA) muscle thickness and length, and multifidus anteroposterior (AP) and mediolateral (ML) thickness during each condition compared to rest (condition one). Data are mean percent change (standard deviation). *P < 0.05, ^†^P < 0.01, ^‡^P < 0.001 compared to rest. Conditions: 2, stretch tall only; 3, stretch tall plus arm extension; 4, stretch tall plus arm extension and thoracic cue; 5, arm extension only; 6, arm extension and thoracic cue.

When compared to rest, contraction of the transversus abdominis (P < 0.001 on repeated measures ANOVA) and multifidus (P < 0.001 on repeated measures ANOVA; Table 1) occurred, but this varied depending on condition (Fig. 3). Transversus abdominis contraction was greatest in the ‘stretch tall plus arm extension’ (length, − 15%, P < 0.001; thickness, + 19%, P < 0.001) and ‘stretch tall plus arm extension and thoracic cue’ (length, − 16%, P < 0.001; thickness, + 18%, P < 0.001) conditions. Transversus abdominis contraction was also apparent, albeit to a lesser magnitude, in the ‘stretch tall only’ (length, − 8.0%, P = 0.019; thickness, + 18%, P < 0.001) and ‘arm extension and thoracic cue’ (thickness only, + 7.8%, P = 0.035) conditions. The contraction of multifidus was the greatest in the ‘arm extension and thoracic cue’ (anteroposterior, + 3.0%, P = 0.001; mediolateral, − 4.2%, P < 0.003) and ‘stretch tall plus arm extension and thoracic cue’ (anteroposterior, + 6.0%, P < 0.001; mediolateral, − 2.1%, P = 0.022) conditions. ‘Stretch tall only’ and ‘stretch tall plus arm extension’ also resulted in increased anteroposterior thickness of 4.4% (P < 0.001) and 5.2% (P < 0.001), respectively, but neither affected mediolateral thickness. Moreover, ‘arm extension only’ led to a 2.8% (P < 0.001) decreased mediolateral thickness, but no change to anteroposterior thickness.

**Table 1:** Transversus abdominis mean muscle thickness and peak muscle length and multifidus peak anteroposterior and mediolateral thickness during each condition and compared to rest (condition one).

Parameter	Condition						RM ANOVA
	1	2	3	4	5	6	
**Transversus abdominis thickness**							
Whole muscle	4.0 (1.1)	4.7 (1.1)^‡^	4.8 (1.0)^‡^	4.7 (1.2)^‡^	4.2 (1.1)	4.3 (1.1)*	P < 0.001
Upper (L1–2)	4.5 (1.1)	5.4 (1.5)^†^	5.2 (1.2)^†^	5.0 (1.0)*	4.7 (1.2)	4.5 (0.9)	P < 0.001
Middle (L3–4)	3.8 (1.2)	4.5 (1.2)^‡^	4.6 (1.1)^‡^	4.6 (1.2)^‡^	4.0 (1.1)	4.3 (1.1)^†^	P < 0.001
Lower (L5–S1)	4.2 (1.5)	4.8 (1.3)^‡^	5.0 (1.4)^‡^	5.0 (1.5)^‡^	4.3 (1.3)	4.5 (1.4)	P < 0.001
**Transversus abdominis length**							
Whole muscle	61.1 (17.9)	56.2 (18.1)*	52.1 (17.2)^‡^	51.2 (18.3)^‡^	59.0 (19.5)	58.3 (19.0)	P = 0.002
Upper (L1–2)	80.2 (15.4)	75.4 (16.8)	76.8 (15.1)	72.8 (13.9)*	87.6 (24.8)	78.9 (20.4)	P = 0.866
Middle (L3–4)	64.9 (20.2)	57.6 (19.7)^‡^	53.8 (19.1)^‡^	54.1 (20.4)^‡^	61.3 (20.0)	62.7 (20.3)	P < 0.001
Lower (L5–S1)	34.8 (9.2)	31.8 (7.9)*	30.7 (8.2)^†^	30.1 (8.2)^‡^	36.1 (9.6)	35.5 (11.5)	P < 0.001
**Multifidus anteroposterior thickness**							
Whole muscle	27.3 (4.1)	28.5 (4.1)^‡^	28.7 (4.1)^‡^	29.0 (4.1)^‡^	27.7 (4.1)	28.1 (4.2)^†^	P < 0.001
Upper (L1–2)	21.4 (3.9)	22.0 (4.0)*	22.2 (4.1)^†^	22.2 (4.0)^†^	21.8 (4.0)	21.4 (4.0)	P = 0.008
Middle (L3–4)	30.7 (5.0)	32.6 (5.0)^‡^	32.9 (5.1)^‡^	33.6 (5.1)^‡^	31.4 (4.9)*	32.1 (5.4)^†^	P < 0.001
Lower (L5–S1)	36.0 (4.4)	36.6 (4.4)	36.3 (4.3)	36.2 (4.3)	35.4 (4.4)	35.2 (4.6)	P < 0.001
**Multifidus mediolateral thickness**							
Whole muscle	24.1 (3.1)	24.2 (3.0)	23.9 (3.1)	23.6 (3.1)*	23.5 (3.0)^‡^	23.1 (3.0)^‡^	P < 0.001
Upper (L1–2)	16.8 (2.5)	17.2 (2.7)	16.9 (2.5)	17.0 (2.5)	17.1 (2.4)	17.0 (2.4)	P = 0.754
Middle (L3–4)	24.6 (3.6)	24.4 (3.5)	24.1 (3.6)*	24.1 (3.7)*	24.5 (3.7)	23.8 (3.7)^†^	P = 0.014
Lower (L5–S1)	28.4 (6.1)	28.5 (6.0)	28.9 (6.1)	29.7 (6.1)^†^	29.5 (6.0)^†^	29.7 (6.1)^†^	P = 0.024

Data are mean (standard deviation) in mm. *P < 0.05, ^†^P < 0.01, ^‡^P < 0.001 compared to rest (condition one). Conditions: 2, stretch tall only; 3, stretch tall plus arm extension; 4, stretch tall plus arm extension and thoracic cue; 5, arm extension only; 6: arm extension and thoracic cue. *RM ANOVA* Repeated measures analysis of variance.

### Regional differences in muscle contraction

**Figure 4:**
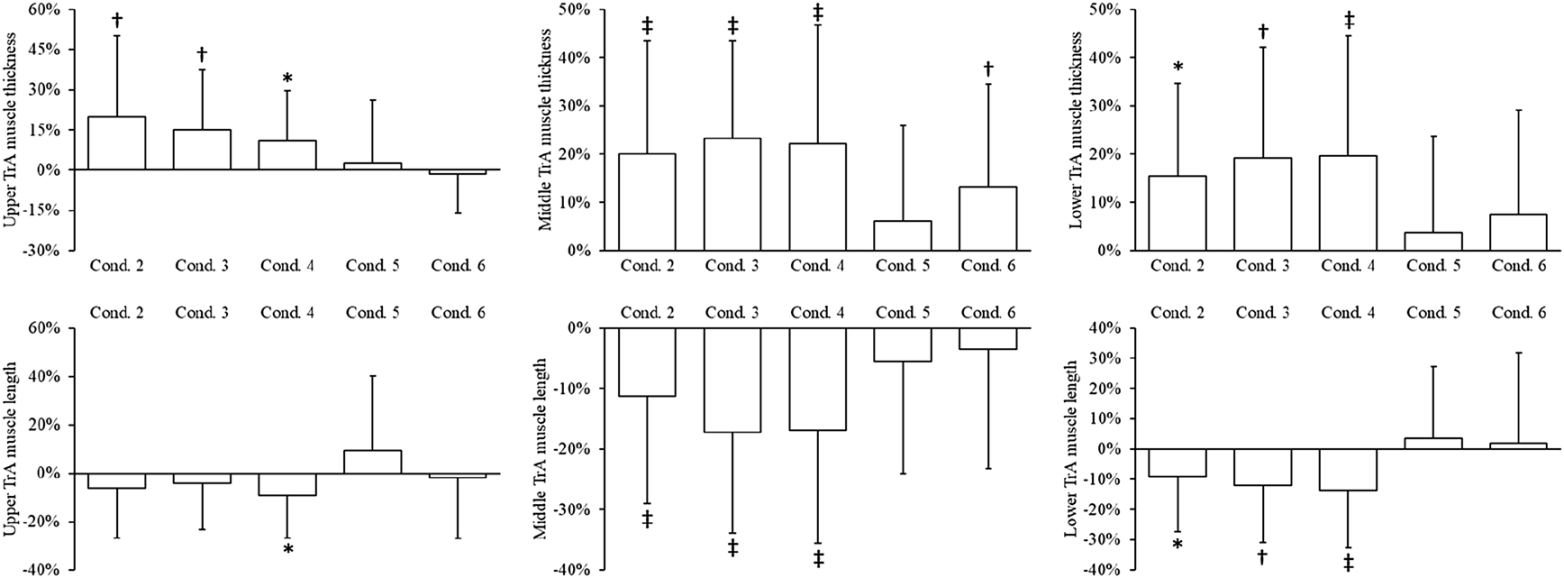
Changes in upper, middle and lower transversus abdominis (TrA) muscle thickness and length during each condition compared to rest (condition one). Data are mean percent change (standard deviation). *P < 0.05, ^†^P < 0.01, ^‡^P < 0.001 compared to rest. Conditions: 2, stretch tall only; 3, stretch tall plus arm extension; 4, stretch tall plus arm extension and thoracic cue; 5, arm extension only; 6, arm extension and thoracic cue.

**Figure 5:**
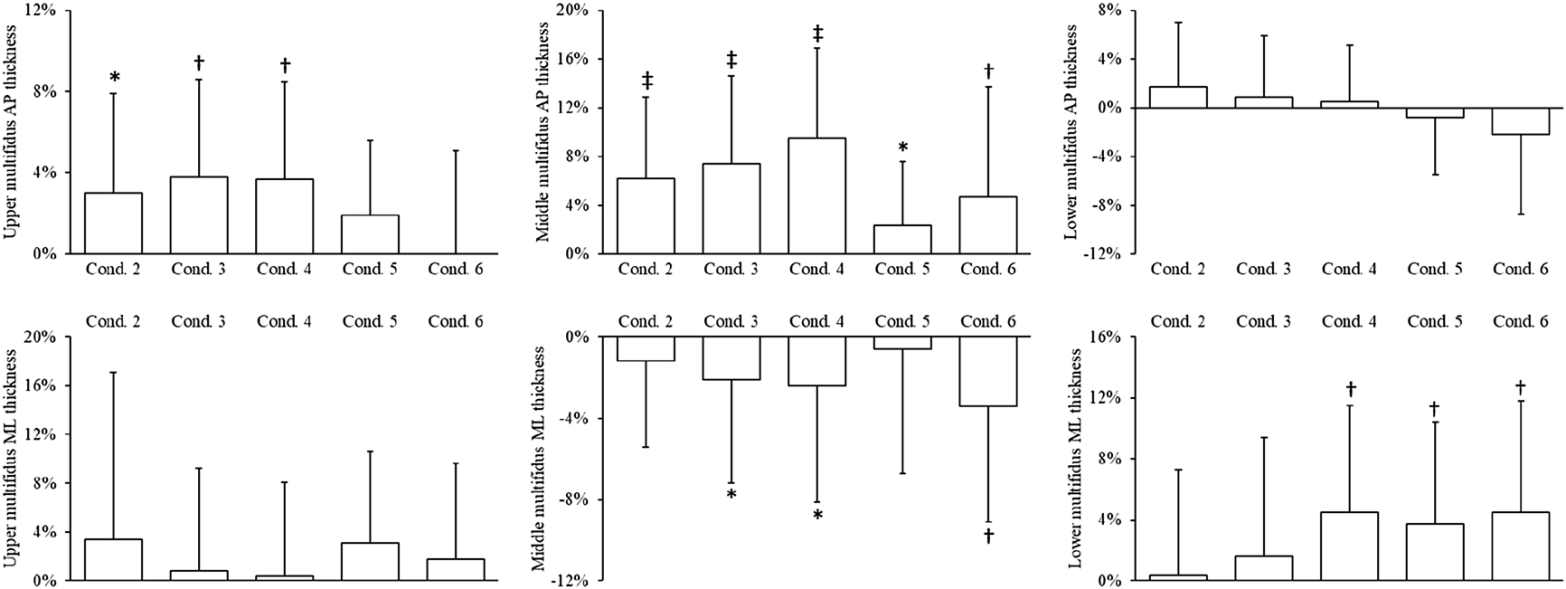
Changes in upper, middle and lower multifidus anteroposterior (AP) and mediolateral (ML) thickness during each condition compared to rest (condition one). Data are mean percent change (standard deviation). *P < 0.05, ^†^P < 0.01, ^‡^P < 0.001 compared to rest. Conditions: 2, stretch tall only; 3, stretch tall plus arm extension; 4, stretch tall plus arm extension and thoracic cue; 5, arm extension only; 6, arm extension and thoracic cue.

Transversus abdominis (Fig. 4) and multifidus (Fig. 5) contraction also varied regionally (Table 1). Contraction of the upper transversus abdominis was greatest in the ‘stretch tall plus arm extension and thoracic cue’ condition (length, − 9.2%, P = 0.035; thickness, + 11%, P = 0.021), whereas the ‘stretch tall plus arm extension’ condition (length, − 17%, P < 0.001; thickness, + 23%, P < 0.001) led to the greatest contraction of the middle region (Fig. 4). The ‘stretch tall plus arm extension and thoracic cue’ condition (length, − 14%, P < 0.001; thickness, + 20%, P < 0.001) also resulted in the greatest contraction of the lower portion of transversus abdominis (Fig. 4).

Regional contraction of transversus abdominis varied significantly in the conditions where arm extension was performed. Compared to upper transversus abdominis, middle region length decreased by 23% (P = 0.002) and 16% (P = 0.002) in the ‘stretch tall plus arm extension’ and ‘stretch tall plus arm extension and thoracic cue’ respectively, and thickness increased by 17% (P < 0.001) in the ‘arm extension and thoracic cue’ condition. Furthermore, lower transversus abdominis thickness was 12% (P = 0.016) greater than that observed in upper transversus abdominis in the ‘arm extension and thoracic cue’ condition.

The upper region of multifidus showed greatest contraction in the ‘stretch tall only’ condition (anteroposterior only, + 3.8%, P = 0.009), whereas the ‘stretch tall plus arm extension and thoracic cue’ condition (anteroposterior, + 9.5%, P < 0.001; mediolateral, − 2.4%, P = 0.042) led to the greatest contraction of the middle region of the muscle (Fig. 5). The lower regions of the multifidus demonstrated the greatest contraction in the ‘stretch tall plus arm extension and thoracic cue’ (anteroposterior only, + 4.5%, P = 0.001) and ‘arm extension and thoracic cue’ (anteroposterior only, + 4.5%, P = 0.002) conditions (Fig. 5).

Regional differences in multifidus contraction were also observed in conditions that involved arm extension. Specifically, the ‘stretch tall plus arm extension’ condition resulted in 3.5% (P = 0.048) greater increase in middle multifidus anteroposterior thickness than upper lumbar multifidus. In the ‘stretch tall plus arm extension and thoracic cue’ condition, a 6.2% increase (P = 0.001) of middle lumbar multifidus anteroposterior thickness was seen compared to other regions. Finally in the ‘stretch tall plus arm extension and thoracic cue’ condition, contraction of both the middle (mediolateral thickness, − 4.7%, P = 0.003; anteroposterior thickness, + 4.7%, P = 0.009) and lower (mediolateral thickness, − 3.7%, P = 0.019; anteroposterior thickness, + 4.3%, P = 0.014) portions of lumbar multifidus were greater than in upper lumbar multifidus.

## Discussion

The main findings from this study were that transversus abdominis contraction was greatest when axial loading through the length of the body and arms was applied, whereas increased multifidus contraction was most markedly achieved with arm extension and a verbal cue aimed at activating the thoracic core (‘thoracic cue’). Moreover, the loading of the head and feet axially with arm extension and the ‘stretch tall’ and ‘thoracic’ cues (condition four) led to the greatest regional (upper, middle and lower) contraction of transversus abdominis. Conversely, multifidus contraction was less consistent across conditions, although the middle lumbar portion of the multifidus muscle was influenced by the axial loading and postural cues.

Our study demonstrated that the exercise approach examined was capable of automatically activating transversus abdominis and multifidus. Given the sizeable atrophy of transversus abdominis (18%)^10^ and multifidus (14%)^20^ during spaceflight simulation (i.e. bed-rest), our findings are of potential clinical relevance to astronauts and future space-faring humans. Specifically, atrophy of muscles responsible for lumbar spine stability, such as transversus abdominis^6^ and multifidus^4^, were proposed as a potential mechanism to explain the increased prevalence of intervertebral disc herniation following spaceflight^2^. Retention of muscle mass and function after spaceflight (or simulation) is better when exercises target specific muscle groups of interest^21^. The findings of the current study support the notion of prospectively examining the efficacy of exercise training with this approach on maintaining ‘core’ muscle function and mass in spaceflight-simulation.

In Earth’s 1-g environment, a range of exercise approaches can activate transversus abdominis and multifidus, although the most effective modality remains debated^22^. Common exercise modalities include those centred on core stability, ball/devices and free weight^22^. A systematic review that examined electromyography activity of transversus abdominis and multifidus during various modalities of exercise in healthy adults concluded that free weights led to the greatest activation of multifidus, whereas no specific modality over another led to increased activation of transversus abdominis^22^. Given the use of free weights involves external resistance and is gravity-dependent, these modalities, unlike the approach examined in our study, are less likely feasible in a microgravity environment.

Structurally, transversus abdominis has been shown to differ in fascicle orientation, thickness and length between upper, middle and lower regions^23^. The middle region attaches to the lumbar spine via the thoracolumbar fascia and is considered to play a greater role in lumbar spine stability^23^. Conversely, the upper and lower regions may play a greater role in stabilizing, respectively, the rib cage (along with involvement in respiration) and the sacroiliac joints^23^. Differential activation of these different portions of this muscle occurs during movement, in particular with the activation of the upper portion differing to that of the middle and lower portions^24,25^.

We observed regional differences in the contraction of the transversus abdominis muscle in exercises that incorporated arm extension. Specifically, middle and lower transversus abdominis showed greater extents of contraction than the upper portion of this muscle in these manoeuvres. This effect was most consistent, statistically, in the middle portion of transversus abdominis. Although the anatomical locations of our MRI measures are not directly comparable those in prior work using fine-wire electromyography, our findings of differential contraction are consistent with the prior studies^24,25^. Our findings also imply that loading of the spine via compression of the arm, with or without axial loading of the body, leads to greater contraction of the middle and lower portions of the transversus abdominis muscle, which is thought to be important for stabilization of the lumbosacral spine.

The findings of the current study may have clinical relevance. Training of the ‘core muscles’, transversus abdominis and lumbar multifidus, is often implemented in the treatment of low back pain and a meta-analysis showed that this kind of exercise can reduce chronic lower back pain^26^. The exercise approaches studied here may represent an alternative form of activating this musculature in the treatment of back pain. Further studies would need to examine efficacy of this exercise approach compared to other forms of training for the ‘core’ musculature.

Our study was strengthened by the use of MRI with a, for this kind of MRI study, large sample size and the blinded nature of assessment. However, the most notable limitation was the cross-sectional design. Whilst we were able to establish the exercise approach examined could activate transversus abdominis and multifidus, the feasibility of translating this into a prospective exercise training model, as well as the potential efficacy, remains to be examined. Additionally, these data were collecting in a 1-g environment with endurance-trained participants, rather than in microgravity with astronauts. Accessing this collective is challenging, hence why we targeted a population with similar physical fitness characteristics, age and gender proportions (6:4, male:female) as the United States NASA Astronaut Corps. The loading levels during the test were set to standardise the testing between subjects. We cannot comment on the percentage of maximum voluntary contraction of transversus abdominis or lumbar multifidus. A further limitation was that we separated the muscles into different regions based on the anatomical level at the lumbar spine. For transversus abdominis, this may not correspond 1:1 to the anatomical divisions of this muscle.

In conclusion, this study demonstrated proof-of-principle for a novel exercise approach capable of automatically activating transversus abdominis and multifidus, via weight bearing through the spine and limbs. Given these findings, determining the feasibility and efficacy of an exercise training program based on this approach for microgravity environments, as well as whether similar observations occur in symptomatic populations, is warranted.
